# Imaging Heterogeneity in Atypical AD

**DOI:** 10.1002/alz70856_099056

**Published:** 2025-12-24

**Authors:** Rosaleena Mohanty, Sophia Wheatley, Giulia Lorenzon, Konstantinos Poulakis, Anna Inguanzo, Eric Westman

**Affiliations:** ^1^ Division of Clinical Geriatrics, Center for Alzheimer Research, Department of Neurobiology, Care Sciences and Society, Karolinska Institutet, Stockholm, Sweden

## Abstract

**Background:**

Neuroimaging has highlighted large biological heterogeneity in Alzheimer's disease (AD). Heterogeneity presenting as selective regional vulnerability has been conceptualized as a combination of **typicality** and **severity** [Ferreira et al., 2020], allowing identification of AD subtypes: typical AD, limbic predominant, cortical predominant and minimal atrophy. Under‐explored questions include: (a) how do subtypes compare across various neuroimaging modalities? (b) how do copathologies contribute to subtype manifestation? (c) can subtypes converge over time? (d) can subtypes track response to treatment? We address these within the revised criteria for AD [Jack et al., 2024].

**Method:**

We examined AD subtypes by typicality (medial temporal‐to‐neocortical biomarker ratio) and severity (global mean of biomarker) with an up‐to‐date review and new evidence on atrophy (MRI), hypometabolism (FDG PET), tau pathology (tau PET) across cohorts (ADNI, Japanese‐ADNI, AIBL, AddNeuroMed, Hippocampus Study). We discuss results in relation to Aβ, tau, neurodegeneration, cerebrovascular and α‐synuclein from the revised criteria for AD.

**Result:**

Typicality and severity effectively captured subtypes in Aβ+ cases exhibiting typical AD, limbic predominant, cortical predominant or minimally affected regional patterns in MRI, FDG PET and tau PET. Addressing our questions: (a) subtypes did not translate across modalities – atrophy did not always mirror tau pathology subtypes; subtypes of atrophy and hypometabolism did not overlap in majority of individuals despite both measuring neurodegeneration. (b) for cerebrovascular copathology in AD, we identified two pathways led by arteriolosclerosis or cerebral amyloid angiopathy. Arteriolosclerosis but not α‐synuclein copathology differentially contributed to subtypes of atrophy and hypometabolism. (c) longitudinal subtyping revealed that limbic predominant and minimal atrophy subtypes converge over time following a mediotemporal pathway, distinct from the cortical predominant pathway. In all subtypes, women exhibited earlier hippocampal atrophy and higher white matter abnormalities than men despite stable cognitive decline. (d) subtypes responded differentially to donepezil treatment in mild cognitively impaired individuals ‐ minimal atrophy and cortical predominant atrophy subtypes showed improved benefit compared to typical AD or limbic predominant atrophy subtypes.

**Conclusion:**

Combination of typicality and severity allows for investigation of neurobiological heterogeneity in AD. Accounting for AD subtypes through multimodal and longitudinal subtyping is valuable for personalized diagnosis, prognosis and treatment.

